# Vascular Endothelial Growth Factor in the Circulation in Cancer Patients May Not Be a Relevant Biomarker

**DOI:** 10.1371/journal.pone.0019873

**Published:** 2011-05-26

**Authors:** Tatjana M. H. Niers, Dick J. Richel, Joost C. M. Meijers, Reinier O. Schlingemann

**Affiliations:** 1 Department of Oncology, Academic Medical Center, University of Amsterdam, Amsterdam, The Netherlands; 2 Department of Vascular Medicine, Academic Medical Center, University of Amsterdam, Amsterdam, The Netherlands; 3 Department of Ophthalmology, Academic Medical Center, University of Amsterdam, Amsterdam, The Netherlands; University of Hong Kong, Hong Kong

## Abstract

**Background:**

Levels of circulating vascular endothelial growth factor (VEGF) have widely been used as biomarker for angiogenic activity in cancer. For this purpose, non-standardized measurements in plasma and serum were used, without correction for artificial VEGF release by platelets activated *ex vivo*. We hypothesize that “true” circulating (c)VEGF levels in most cancer patients are low and unrelated to cancer load or tumour angiogenesis.

**Methodology:**

We determined VEGF levels in PECT, a medium that contains platelet activation inhibitors, in citrate plasma, and in isolated platelets in 16 healthy subjects, 18 patients with metastatic non-renal cancer (non-RCC) and 12 patients with renal cell carcinoma (RCC). In non-RCC patients, circulating plasma VEGF levels were low and similar to VEGF levels in controls if platelet activation was minimized during the harvest procedure by PECT medium. In citrate plasma, VEGF levels were elevated in non-RCC patients, but this could be explained by a combination of increased platelet activation during blood harvesting, and by a two-fold increase in VEGF content of individual platelets (controls: 3.4 IU/10^6^, non-RCC: 6.2 IU/10^6^ platelets, p = 0.001). In contrast, cVEGF levels in RCC patients were elevated (PECT plasma: 64 pg/ml vs. 21 pg/ml, RCC vs. non-RCC, p<0.0001), and not related to platelet VEGF concentration.

**Conclusions:**

Our findings suggest that “true” freely cVEGF levels are not elevated in the majority of cancer patients. Previously reported elevated plasma VEGF levels in cancer appear to be due to artificial release from activated platelets, which in cancer have an increased VEGF content, during the blood harvest procedure. Only in patients with RCC, which is characterized by excessive VEGF production due to a specific genetic defect, were cVEGF levels elevated. This observation may be related to limited and selective success of anti-VEGF agents, such as bevacizumab and sorafenib, as monotherapy in RCC compared to other forms of cancer.

## Introduction

Vascular endothelial growth factor (VEGF) is the prime regulator of angiogenesis in tumours [1]–[3]. It is released by cancer cells, hypoxic cells and activated platelets and leukocytes [4]–[8]. The target cells of VEGF are primarily vascular endothelial cells on which it has powerful mitogenic effects via high affinity receptors Flt-1 and KDR/Flk-1 [9], [10]. As VEGF is a soluble diffusible peptide secreted by tumours, its levels in the circulation were proposed to reflect the angiogenic activity of malignancies. Hence in the past years, circulating levels of VEGF and other angiogenic factors have been widely studied as surrogate markers of angiogenic activity and prognosis in cancer patients, for monitoring treatment response and for detection of early relapse [11].

For this purpose, VEGF levels determined in serum or plasma were used. However, serum contains high levels of VEGF due to release by activated platelets during clotting [5]–[7]. Therefore, VEGF levels in serum, which correlate closely with blood platelet count [12], do not reflect the actual circulating concentration of VEGF in vivo. In citrate or EDTA plasma, where less platelet activation and subsequent VEGF release is expected than in serum, VEGF levels were found to be still higher in cancer patients than in controls and this was interpreted as a reflection of higher levels of VEGF in the circulation and higher angiogenic activity [13]–[15]. But also by these authors artificial release of VEGF from platelets, or altered behaviour of platelets in cancer patients, was not excluded as a source of increased VEGF levels in the plasma samples.

The aim of this study was to investigate the possibility that artificial VEGF release by platelets is the main source of VEGF in plasma samples, and that circulating levels of VEGF in cancer patients are low and even unrelated to cancer load or angiogenic activity. For this purpose, we determined plasma levels of VEGF with or without inhibition of platelet activation, and quantified VEGF release from platelets in vitro, in control persons without cancer, patients with metastatic non-renal cell carcinoma patients and patients with metastatic renal cell carcinoma.

## Materials and Methods

### Patients and volunteers

We obtained venous blood from 16 healthy volunteers (Controls) and from 30 cancer patients, separated in two patients groups: 18 patients with metastatic non-RCC (Non-RCC) and 12 patients with metastatic RCC (RCC). RCC was characterized by high intra-tumour VEGF production by mutations in the von Hippel-Lindau tumour suppressor gene with consequent unlimited activity of the hypoxia inducible factor 1α (HIF1α) causing high VEGF transcription [16]–[20].

We investigated VEGF levels in citrate plasma, PECT plasma and in platelets. The characteristics of volunteers and patients are as follows: healthy controls (Controls) median age, 53 [48–60]; cancer patients (Non-RCC-group); median age, 62 [57–72]; with cancer of the pancreas (7), cholangio (3), Papilli of Vater (2), esophagus (1), colon and rectum (2), melanoma (1), ovary(1), breast (1). RCC (RCC-group) median age, 60 [56–65]; with the following prognostic groups: 5 poor-risk, 5 intermediate risk and 2 favourable risk, according to Motzer [21]. All cancer patients had metastatic disease. To discriminate between in vivo platelet activation and artificial in vitro platelet activation during plasma harvest, β-TG and PF4 were measured in all samples. Blood samples used in this study were derived from RCC patients included in the BAYER 43-9006 study (approved by the Medical Ethics Committee (METC 05-262), Academic Medical Center, university of Amsterdam), non-RCC patients not included in a specific study, and volunteers. From all volunteers and patients separate written informed consent was obtained for blood sample harvest and angiogenesis biomarker determinations.

### Plasma preparations

For comparison with standard citrate plasma collection, we employed a method to (maximally) avoid artificial ex vivo platelet activation by blood collection without tourniquet, and harvest in a medium containing a mixture of anticoagulants to which prostaglandin E_1_ and theophylline was added (PECT medium). In addition, we used an important tool to discriminate between in vivo platelet activation and artificial in vitro platelet activation, i.e. measurements of β-thromboglobulin (β-TG) and platelet factor 4 (PF4) levels. Collection in PECT plasma and concomitant use of markers of platelet activation should provide an accurate estimation of the circulating levels of VEGF in vivo.

From each patient or volunteer venous blood was taken with a microperfuser (diameter 1 mm, Microflex, Vycon, Ecouen, France) and divided into different tubes. Plastic (polypropylene) blood collection tubes were filled with 400 µL of a solution containing: prostaglandin E_1_ (94 nM), Na_2_CO_3_ (0.63 mM), EDTA (90 mM) and theophylin (10 mM) (PECT-medium). Blood samples (4 ml) were collected in these PECT tubes in an open system, drop by drop without using a tourniquet to (maximally) avoid platelet activation ex vivo. Blood collected in the PECT tubes was immediately placed on ice (in contrast to the citrate and EDTA blood samples which were kept at room temperature). Platelet-depleted PECT plasma was prepared by spinning for 60 min at 1700 g at 4°C within 1 hour after collection.

Blood was also collected in tubes filled with citrate and in tubes filled with EDTA using a tourniquet (Becton Dickinson Vacutainers Systems, Breda, The Netherlands). The citrate blood samples were centrifuged within 30 minutes for 15 minutes at 1000 g to obtain plasma. EDTA blood drawn in the same manner was used to measure total number of platelets. To measure VEGF, PF4 and β-TG within the platelets, EDTA blood was used in which platelets were destroyed by a combination of Triton (2% Triton X-100), sonication during 15 seconds on ice (microtip, Branson, amplitude 50%) and centrifuging during 5 minutes at 14.000 rpm in a micro-centrifuge. Platelet-depleted PECT plasma was used to measure VEGF, PF4 and β-TG levels. Citrate plasma was used to measure VEGF and PF4 levels.

### Measurements of platelet activation markers and VEGF

PF4 and β-TG concentrations within platelets are similar and upon platelet activation they are released in similar quantities [22],[23]. Because PF4 clearance from plasma is much faster than β-TG clearance (t ½ for PF4 is several minutes and for β-TG>100 minutes) [22],[23] a normal or only slightly elevated PF4 level and high β-TG level suggest in vivo platelet activation, whereas a high β-TG level in combination with a high PF4 level suggests in vitro (ex-vivo) platelet activation. All samples were therefore tested for VEGF, β-TG and PF4 using commercially available sandwich enzyme–linked immunosorbent assays (ELISAs) from Roche (Asserachrom β-TG and Asserachrom PF4; Roche, Mannheim, Germany) and R&D Systems (Quantikine VEGF; R&D Systems, Abingdon, UK).

### Recovery of VEGF

To exclude the possibility that PECT or citrate medium affects the measurement of VEGF levels by ELISA, known concentrations of recombinant human VEGF (standard provided in the assay kit) were added to serum, PECT plasma and citrate plasma samples to produce VEGF concentrations of 250, 62.5, 31.2, 7.8 and 3.9 pg/ml. The samples were diluted with assay buffer and the concentration of VEGF was determined by ELISA.

### Statistical analysis

Statistical analysis was performed with the computer program SPSS version 12.0 (SPSS, Gorinchem, The Netherlands) and with GraphPad Prism software (GraphPad Prism, San Diego, CA, USA). VEGF, PF4 and β-TG levels in healthy volunteers and cancer patients were compared using the Mann-Whitney U-test (unpaired, non-normally distributed groups). Values are presented as median and interquartile range [t25–t75].

## Results

### Plasma and platelet VEGF levels

**Figure 1:**
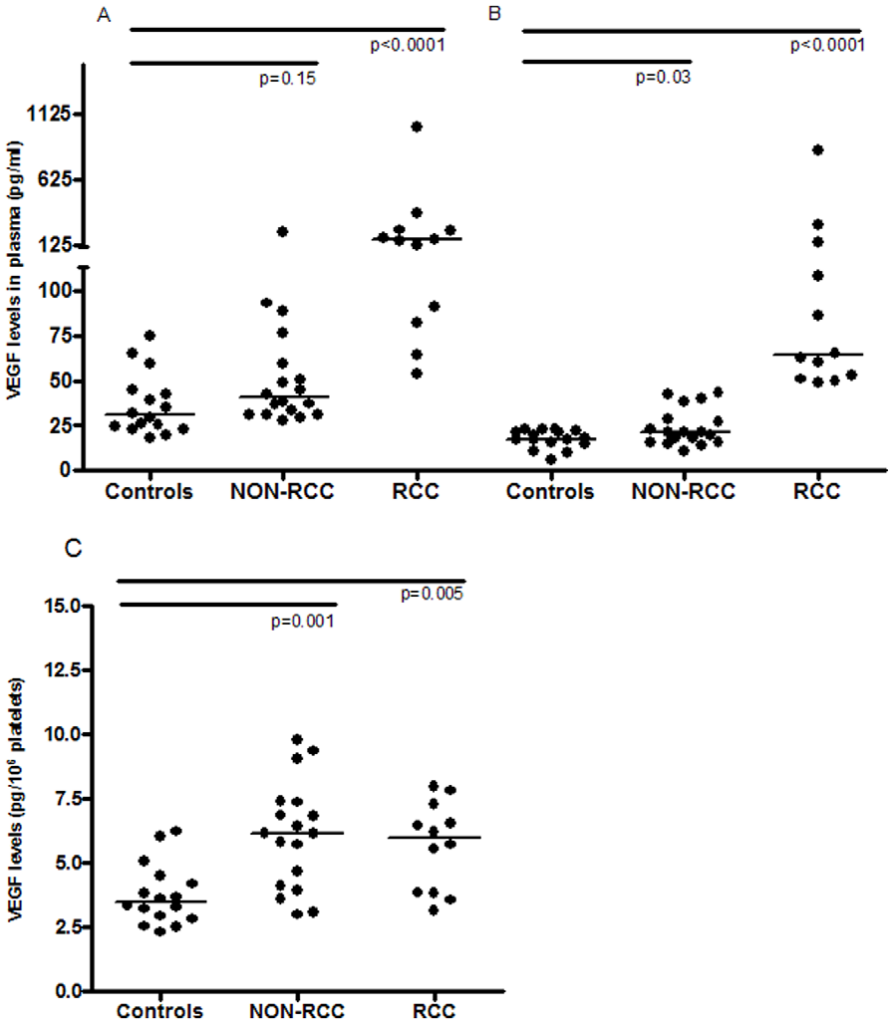
VEGF levels in cancer patients and controls. A) VEGF-levels measured in citrate plasma of cancer patients compared to healthy persons. B) VEGF-levels measured in PECT plasma of cancer patients compared to healthy persons. C) VEGF levels measured in platelets of cancer patients versus healthy persons. Bars represent the medians.

In the three patient groups, VEGF levels in citrate plasma (Figure 1A) were significantly higher than in PECT plasma (Figure 1B). VEGF levels in citrate plasma were significantly higher in both groups of cancer patients compared to healthy controls, but in PECT plasma this was only the case for patients with RCC. The VEGF content of isolated platelets, a well known reservoir for VEGF, was two-fold higher in both cancer patient groups than in controls (Table 1 and Figure 1C).

**Table 1:** VEGF levels in healthy volunteers and cancer patients.

Characteristics	Controls	Non-RCC	RCC	P value Mann Whitney Co. vs. Non-RCC
VEGF–citrate (pg/ml)	30.8 [23.5–44.3]	37.8 [31.2–52.8]	166 [84–238]	0.03
VEGF–PECT (pg/ml)	17.4 [15.2–22.2]	21.0 [16.0–31.5]	64 [52–134]	NS (0.15)
VEGF in platelets (pg/10^6^ platelets)	3.4 [2.8–4.4]	6.2 [4.1–7.4]	6.0 [3.8–7.1]	0.001

Controls; healthy volunteers; Non-RCC; non-renal carcinoma patients; RCC; renal cell carcinoma patients. VEGF levels in median [interquartile range].

In RCC patients, VEGF levels in PECT plasma were considerably higher than in non-RCC patients, whereas the platelet VEGF content was not different between these groups. VEGF levels in citrate plasma, PECT plasma, and platelets in the patients group are shown in Table 1 and Figure 1. These measurements were not affected by the type of medium used, as recovery of VEGF was 100–107% in PECT plasma, 73%–107% in citrate plasma and 78–100% in serum.

### *In vitro* platelet activation

Due to its low half life in vivo, PF4-levels in blood samples are a measure of artificial ex vivo platelet activation. In our study, in both healthy controls and cancer patients PF4 concentrations were 50–100 times higher in standard citrate plasma (collected with tourniquet) than in PECT plasma (collected without tourniquet) (Table 2). This demonstrates that the degree of artificial ex-vivo platelet activation is highly dependent on the method of harvest, and that artificial platelet activation in PECT plasma is low and in citrate plasma samples is high.

**Table 2:** Platelet and platelet activation parameters.

Characteristics	Controls	Non-RCC	P value Mann Whitney Co. vs. Non-RCC
Number of platelets (10^9^/l)	259 [237–278]	278 [225–382]	NS (0.13)
PF4-PECT (IU/ml)	7.7 [5.6–10.3]	14·2 [11.1–87.5]	0.0002
PF4-citrate (IU/ml)	523 [298–738]	657 [283–806]	NS (0.81)
PF4-in platelets (IU/10^6^ platelets)	11·3 [9.8–12.5]	15·2 [13.3–17.2]	0.0005
β-TG-PECT (IU/ml)	37.4 [24.1–46.4]	74·7 [51.4–151.4]	0.0005
β-TG-in platelets (IU/10^6^ platelets)	34.3 [29.6–39.3]	36.9 [30.6–41.4]	NS (0.52)

Controls; healthy volunteers; Non-RCC; non-renal carcinoma patients. VEGF levels in median [interquartile range].

**Figure 2:**
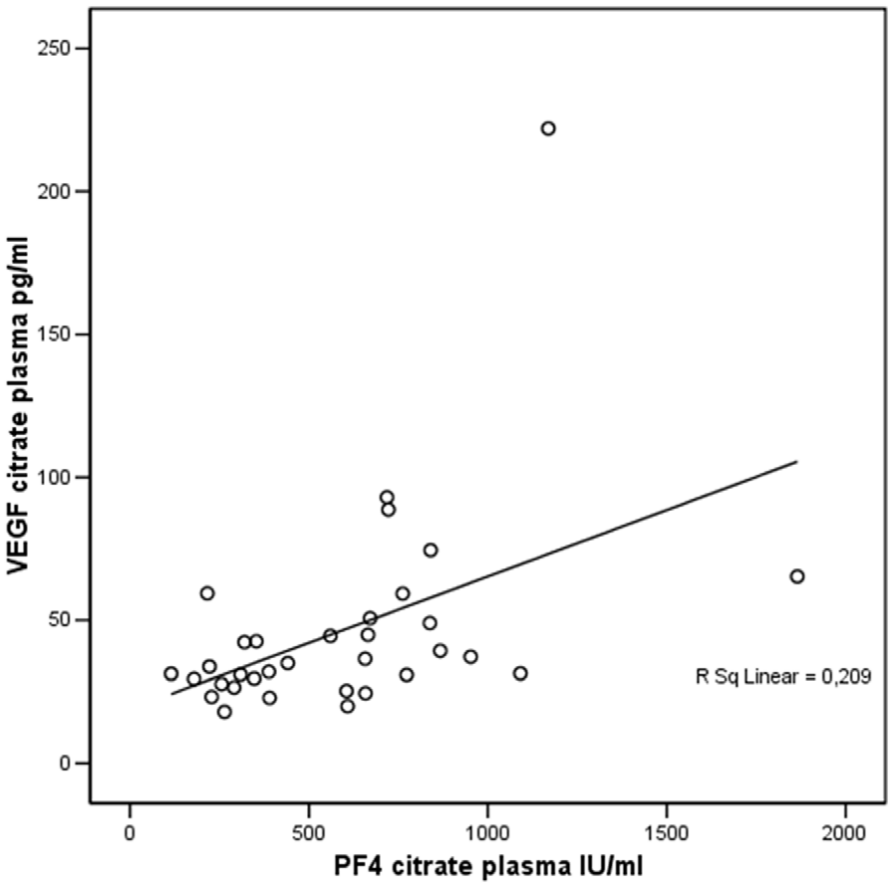
Correlation between VEGF and PF4 in citrate plasma in cancer patients and controls. VEGF and PF4 measured in citrate plasma correlated significantly (r = 0.457, p = 0.008).

As platelet activation is associated with VEGF release, PECT plasma VEGF levels probably reflect the circulating levels of VEGF, while the increased levels of VEGF in citrate plasma are mostly caused by ex vivo platelet activation. This possibility is strongly supported by the significant correlation which we observed between PF4 and VEGF levels in individual citrate plasma samples (Figure 2).

**Figure 3:**
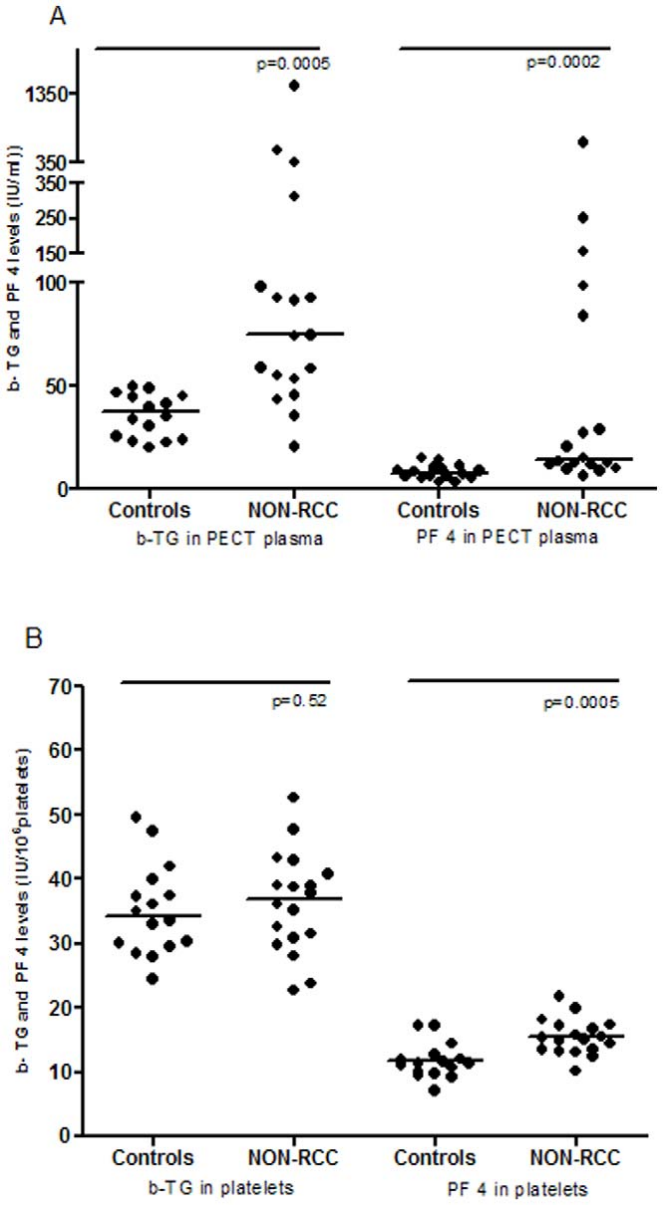
Scatter plot presentation of the distribution of β-TG and PF4. A) β-TG and PF4 measured in PECT plasma of cancer patients compared to controls. B) β-TG and PF4 measured in platelets of cancer patients compared to controls. Bars represent the medians.

In PECT plasma, we found two-fold higher PF4 values in cancer patients compared to healthy controls. This indicates that platelets from cancer patients are more prone to become activated. However, an increased PF4 concentration in individual platelets of cancer patients may also have contributed to the increased PF4 concentration in PECT plasma (Figure 3).

A high β-TG level in combination with a low PF4 level in PECT plasma is an indication of in vivo platelet activation. We were unable to demonstrate such in vivo platelet activation in our patients with cancer, as β-TG in PECT plasma did not show an increase independent of the increase in PF4.

## Discussion

Our study demonstrates that ‘true’ circulating levels of VEGF, as determined by measurements in PECT plasma, are low in the majority of patients with metastatic cancer and that they do not differ significantly from circulating VEGF levels in controls. The elevated VEGF levels in citrate plasma samples correlate with PF4, a marker of ex vivo platelet activation, suggesting that release of VEGF from platelets during the harvest procedure is responsible for the VEGF levels in these samples. In addition, we observed that individual platelets in cancer patients have a two-fold higher content of VEGF compared to healthy controls. Our findings show that VEGF levels in blood samples are highly dependent on the method of collection and platelet VEGF content, and question the relevance of ‘circulating’ VEGF as a biomarker. Our results do not rule out that artificially elevated VEGF levels in citrate plasma samples, as a combined measure of platelet activatibility and platelet VEGF content, could not be a meaningful surrogate marker of certain aspects of tumour biology.

A marked increase in plasma VEGF levels has been observed in various types of cancer employing various collection and determination methods [11]. A number of studies reported a correlation between platelet counts and serum VEGF [24], [25], and higher serum VEGF levels per platelet in cancer patients [26], [27]. This is in line with our findings and the known function of platelets as an important reservoir for VEGF. Platelet-derived VEGF may have an important pathological role in cancer due to thrombin induced platelet activation and subsequent local release of VEGF, inducing vascular permeability, endothelial cell activation and angiogenesis, promoting coagulation and cancer dissemination [4], [28]–[30].

In the present study, we evaluated two different methods of plasma collection for VEGF measurement in healthy controls and cancer patients. The impact of the use of a tourniquet and collection medium (citrate versus PECT) on VEGF levels was demonstrated in healthy controls and cancer patients. No significant differences were detected in PECT VEGF levels between healthy controls and non-RCC cancer patients.

In RCC patients, PECT VEGF levels were significantly higher compared to controls, suggesting that in these patients VEGF is truly elevated in the circulation. RCC is characterized by high intra-tumour VEGF production by homozygous mutations in the von Hippel-Lindau tumour suppressor gene with consequent unlimited activity of the hypoxia inducible factor 1α (HIF1α) causing high VEGF transcription [20]–[26]. In many of these patients, the other cells in the body are heterozygous for the VHL mutation. These genetic aspects may explain why RCC patients have different circulating VEGF levels than the non-RCC patients studied.

Our findings are in line with the selective success of anti-VEGF approaches in the treatment of RCC compared to other types of cancer [31]. Indeed, bevacizumab (Avastin; Genentech, South San Francisco, CA) an antibody that binds to all isoforms of human cVEGF [32] produced a significant prolongation of time to disease progression compared with placebo in patients with RCC [33]. This in contrast with most other tumor types, where bevacizumab is either not effective or had only significantly antitumor efficacy when used in combination with chemotherapy rather than as monotherapy (acting more like chemotherapy-enhancer) [34]. The straightforward explanation for the clinically relevant activity of bevacizumab as a monotherapy in RCC might be the specific roles of defective hypoxic signalling and VEGF overexpression in the pathogenesis of these tumors.

We observed that in vitro platelet activation during the collection procedure contributes to higher citrate plasma VEGF levels. This was demonstrated by the high release of PF4 from platelets in citrate compared to PECT plasma, and the significant positive correlation between PF4 and VEGF levels in individual citrate plasma samples. The two-fold higher levels of PF4 in PECT plasma in cancer patients compared to controls suggest that also with the optimal collection method some platelet activation still occurs, but more importantly this shows that platelets in cancer patients become more easily activated than platelets from healthy controls.

In line with other studies we demonstrated that platelet VEGF content is higher in cancer patients [26], [27], [35]. In the light of our findings in PECT plasma, this suggests that ex vivo release of VEGF by platelets is altered in cancer patients, due to both an increase in platelet VEGF content as well as to a higher activatibility of platelets in cancer patients. Higher platelet VEGF content may originate from increased loading in the bone marrow or result from a VEGF scavenging function of platelets [6], [35], [36]. Such a scavenging function would serve to remove excess VEGF, produced locally in tumour tissues, from the circulation. It is remarkable that in both our patient groups the increase in platelet VEGF content was about two fold compared to controls, despite the much higher VEGF levels in PECT plasma from RCC patients. Therefore, if platelets really have a scavenging function for VEGF, in RCC this scavenging function appears to fail due to excessively high VEGF production, leading to circulating VEGF. In addition, in contrast to circulating free VEGF, VEGF content within platelets may be a meaningful interesting potential biomarker for VEGF and/or angiogenesis activity in cancer patients, which needs further studies.

In conclusion, free VEGF levels are low or absent in the circulation in most cancer patients, with the exception of RCC, a cancer type with excessive VEGF production due to a specific genetic defect. Citrate VEGF levels do not reflect actual circulating VEGF levels but are the result of ex vivo platelet activation and subsequent VEGF release from platelets which have an increased VEGF content in cancer patients. Elevated citrate VEGF levels in cancer patients, which have widely been used as a biomarker of tumour angiogenesis, are caused by artefacts and altered platelet behaviour associated with the systemic disease.

## References

[1] Folkman J (1995). Seminars in Medicine of the Beth Israel Hospital,Boston. Clinical applications of research on angiogenesis.. N.Engl.J.Med.

[2] Veikkola T, Alitalo K (1999). VEGFs, receptors and angiogenesis.. Semin.Cancer Biol.

[3] Shibuya M, Claesson-Welsh L (2006). Signal transduction by VEGF receptors in regulation of angiogenesis and lymphangiogenesis.. Exp.Cell Res.

[4] Mohle R, Green D, Moore MA, Nachman RL, Rafii S (1997). Constitutive production and thrombin-induced release of vascular endothelial growth factor by human megakaryocytes and platelets.. Proc.Natl.Acad.Sci.U.S.A.

[5] Banks RE, Forbes MA, Kinsey SE, Stanley A, Ingham E (1998). Release of the angiogenic cytokine vascular endothelial growth factor (VEGF) from platelets: significance for VEGF measurements and cancer biology.. Br.J.Cancer.

[6] Verheul HM, Hoekman K, Luykx-de Bakker S, Eekman CA, Folman CC (1997). Platelet: transporter of vascular endothelial growth factor.. Clin.Cancer Res.

[7] Gunsilius E, Petzer A, Stockhammer G, Nussbaumer W, Schumacher P (2000). Thrombocytes are the major source for soluble vascular endothelial growth factor in peripheral blood.. Oncology.

[8] Webb NJ, Myers CR, Watson CJ, Bottomley MJ, Brenchley PE (1998). Activated human neutrophils express vascular endothelial growth factor (VEGF).. Cytokine.

[9] Witmer AN, Vrensen GF, Van Noorden CJ, Schlingemann RO (2003). Vascular endothelial growth factors and angiogenesis in eye disease.. Prog.Retin.Eye Res.

[10] Witmer AN, Dai J, Weich HA, Vrensen GF, Schlingemann RO (2002). Expression of vascular endothelial growth factor receptors 1, 2, and 3 in quiescent endothelia.. J.Histochem.Cytochem.

[11] Longo R, Gasparini G (2007). Challenges for patient selection with VEGF inhibitors.. Cancer Chemother.Pharmacol.

[12] Salgado R, Vermeulen PB, Benoy I, Weytjens R, Huget P (1999). Platelet number and interleukin-6 correlate with VEGF but not with bFGF serum levels of advanced cancer patients.. Br.J.Cancer.

[13] Hyodo I, Doi T, Endo H, Hosokawa Y, Nishikawa Y (1998). Clinical significance of plasma vascular endothelial growth factor in gastrointestinal cancer.. Eur.J.Cancer.

[14] Davies MM, Jonas SK, Kaur S, Allen-Mersh TG (2000). Plasma vascular endothelial but not fibroblast growth factor levels correlate with colorectal liver mestastasis vascularity and volume.. Br. J. Cancer.

[15] Yoshikawa T, Tsuburaya A, Kobayashi O, Sairenji M (2000). Plasma concentrations of VEGF and bFGF in patients with gastric carcinoma.. Cancer Lett.

[16] Gnarra JR, Zhou S, Merrill MJ, Wagner JR, Krumm A (1996). Post-transcriptional regulation of vascular endothelial growth factor mRNA by the product of the VHL tumor suppressor gene.. Proc.Natl.Acad.Sci.U.S.A.

[17] Iliopoulos O, Levy AP, Jiang C, Kaelin WG, Goldberg MA (1996). Negative regulation of hypoxia-inducible genes by the von Hippel-Lindau protein.. Proc.Natl.Acad.Sci.U.S.A.

[18] Mukhopadhyay D, Knebelmann B, Cohen HT, Ananth S, Sukhatme VP (1997). The von Hippel-Lindau tumor suppressor gene product interacts with Sp1 to repress vascular endothelial growth factor promoter activity.. Mol.Cell Biol.

[19] Kapitsinou PP, Haasse PP (2008). The VHL tumor suppressor and HIF; insights from genetic studies in mice.. Cell Death and differentiation.

[20] Nyhan MJ, O'Sullivan GC, McKenna SL (2008). Role of the VHL (von Hippel-Lindau) gene in renal cancer: a multifunctional tumour suppressor.. Biochemical Society transactions.

[21] Motzer RJ, Mazumdar M, Bacik J, Berg W, Amsterdam A (1999). Survival and prognostic stratification of 670 patients with advanced renal cell carcinoma.. J.Clin.Oncol.

[22] Kaplan KL, Owen J (1981). Plasma levels of beta-thromboglobulin and platelet factor 4 as indices of platelet activation in vivo.. Blood.

[23] Kaplan KL, Owen L, Harker LA (1986). Plasma Levels of platelet secretory proteins. Crit Rev Oncol.. Hematol.

[24] Caine GJ, Lip GY, Blann AD (2004). Platelet-derived VEGF, Flt-1, angiopoietin-1 and P-selectin in breast and prostate cancer: further evidence for a role of platelets in tumour angiogenesis.. Ann. Med.

[25] Werther K, Christensen IJ, Nielsen HJ (2002). Determination of vascular endothelial growth factor (VEGF) in circulating blood: significance of VEGF in various leucocytes and platelets.. Scand. J. Clin. Lab Invest.

[26] Kusumanto YH, Dam WA, Hospers GA, Meijer C, Mulder NH (2003). Platelets and granulocytes, in particular the neutrophils, form important compartments for circulating vascular endothelial growth factor.. Angiogenesis.

[27] Salven P, Orpana A, Joensuu H (1999). Leukocytes and platelets of patients with cancer contain high levels of vascular endothelial growth factor.. Clin Cancer Res.

[28] Verheul HM, Pinedo HM (1998). Tumor Growth: A Putative Role for Platelets?. Oncologist.

[29] Verheul HM, Jorna AS, Hoekman K, Broxterman HJ, Gebbink MF (2000). Vascular endothelial growth factor-stimulated endothelial cells promote adhesion and activation of platelets.. Blood.

[30] Verheul HM, Hoekman K, Lupu F, Broxterman HJ, van der Valk P (2000). Platelet and coagulation activation with vascular endothelial growth factor generation in soft tissue sarcomas.. Clin.Cancer Res.

[31] Rini BI, Small EJ (2005). Biology and clinical development of vascular endothelial growth factor targeted therapy in renal cell carcinoma.. J Clin Oncol.

[32] Presta LG, Chen H, O Çonnor SJ (1997). Humanization of an antivascular endothelial growth factor monoclonal antibody for the therapy of solid tumors and other disorders Cancer Res.

[33] Yang JC, Haworth L, Sherry RM (2003). A randomized trial of bevacizumab, an anti-vascular endothelial growth factor antibody, for metastatic renal cancer.. N Engl J Med:.

[34] Grothey A, Ellis LM (2008). Targeting angiogenesis driven by endothelial growth factors using antibody based therapy.. Cancer Journal.

[35] Klement GL, Yip TT, Cassiola F, Kikuchi L, Cervi D (2009). Platelets actively sequester angiogenesis regulators.. Blood.

[36] George ML, Eccles SA, Tutton MG, Abulafi AM, Swift RI (2000). Correlation of plasma and serum vascular endothelial growth factor levels with platelet count in colorectal cancer: clinical evidence of platelet scavenging?. Clin.Cancer Res.

